# New Journals

**Published:** 1881-10-20

**Authors:** 


### *NEW JOURNALS.*

Dr. Billings, in a paper read before the International Medical Congress, says that twenty-three new medical journals ceased to exist in 1879, and yet sixty new ones appeared. The great majority of the new journals which are coming out place their subscription prices at figures so low that failure, sooner or later, is inevitable. The result is that serious injury is inflicted upon journalism as a business, while medical literature is in no important degree promoted,

